# Alternative moderne în managementul obezităţii

**Published:** 2014

**Authors:** Popa Teodora Alexandra, Ladea Maria, Grosu Larisa Silvia

**Affiliations:** * Spitalul Clinic de Psihiatrie „Prof. Dr. Al. Obregia”, București, România, Spitalul Clinic de Urgență “Sf. Pantelimon”, București, România

**Rezumat**

România se clasează în primele zece locuri în Europa privind numărul persoanelor supraponderale. Rata obezităţii a crescut progresiv în ultimii 30 de ani, ceea ce impune dezvoltarea şi aplicarea la scală largă a unor măsuri de prevenire, tratare şi sprijin. Programele de autoajutorare deservesc mult mai mulţi oameni comparativ cu tratamentele medicale ori chirurgicale împotriva obezităţii.

Există puţine date privind eficienţa programelor nonmedicale adresate scăderii în greutate. Un studiu recent arată că programele bazate pe grupuri suportive au avut rezultate superioare comparativ cu dietele individuale.

Programele supervizate de medici combină modificarea stilului de viaţă cu dieta hipocalorică. Eficacitatea şi siguranţa acestora au fost analizate, determinând o pierdere de aproximativ 20% din greutatea iniţială în primele luni. În lipsa unui program de menţinere a greutăţii, indivizii recâştigă din greutatea pierdută.

Programele accesibile pe internet oferă sprijin nelimitat, discuţii online şi acces permanent la informaţii despre diete, exerciţii fizice şi modificarea stilului de viaţă.

Studii care să stabilească efectele pe termen scurt şi lung, publicarea datelor despre costurile şi riscurile asociate acestor programe sunt necesare în atragerea şi menţinerea indivizilor în program şi pentru luarea unei decizii informate asupra metodei adecvate de scădere în greutate.

**Cuvinte-cheie:** dieta, obezitate, management

